# Full regeneration of descending corticotropin-releasing hormone axons after a complete spinal cord injury in lampreys

**DOI:** 10.1016/j.csbj.2022.10.020

**Published:** 2022-10-18

**Authors:** Laura González-Llera, Daniel Sobrido-Cameán, Gabriel N. Santos-Durán, Antón Barreiro-Iglesias

**Affiliations:** Department of Functional Biology, CIBUS, Faculty of Biology, Universidade de Santiago de Compostela, 15782 Santiago de Compostela, Spain

**Keywords:** CRH, Descending axons, Spinal cord injury, Axon regeneration, Immunohistochemistry, Lamprey

## Abstract

Sea lampreys are a vertebrate model of interest for the study of spontaneous axon regeneration after spinal cord injury (SCI). Axon regeneration research in lampreys has focused on the study of giant descending neurons, but less so on neurochemically-distinct descending neuronal populations with small caliber axons. Corticotropin-releasing hormone (CRH) is a neuropeptide that regulates the stress response or locomotion. CRH is also a neuropeptide of interest in the SCI context because descending CRHergic projections from the Barrington’s nucleus control micturition behavior in mammals. Recent work from our group revealed that in sea lampreys the CRHergic innervation of the spinal cord is only of descending origin. Thus, the lack of intrinsic CRH spinal cord neurons provides the opportunity to analyze the regeneration of this descending system by using immunofluorescence methods. Here, we used an antibody against the sea lamprey mature CRH peptide, confocal microscopy, lightning adaptive deconvolution, and ImageJ to analyze the regenerative capacity of the descending CRH-immunoreactive (-ir) axons of larval sea lampreys after a complete SCI at the level of the fifth gill. At 10 weeks post-lesion, when behavioral analyses showed that injured animals had recovered normal appearing locomotion, our results revealed a full recovery of the number of CRH-ir profiles (axons) at the level of the sixth gill. Thus, the CRH descending axons of lampreys fully regenerate after a complete SCI. Our study provides a new model to study spontaneous and successful axonal regeneration in a specific neuronal type with small caliber axons by using simple immunohistochemical methods.

## Highlights

• In lampreys, all spinal cord CRHergic axons are of descending origin.
• CRHergic axons fully regenerate at the level of the 6th gill after a complete SCI.
• Regenerated axons re-establish synaptic contacts with targets below the injury.

## Graphical abstract

**Figure:**
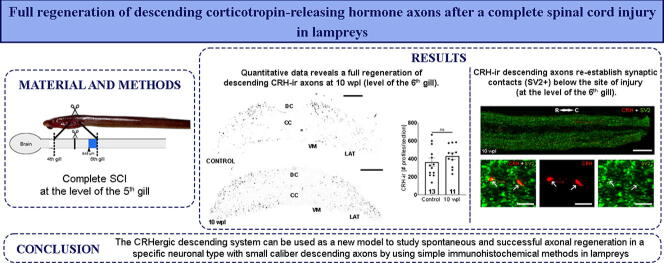

## 1 Introduction

In recent years there has been a surge of interest to study animal models of spontaneous and successful regeneration after a spinal cord injury (SCI). For example, many fish species, in contrast to most mammals (including humans), can spontaneously regenerate descending axons after a SCI (e.g., zebrafish: [1]; goldfish: [2], [3]; lampreys: [4]). One of the animal models for the study of successful and spontaneous spinal cord regeneration is the sea lamprey *Petromyzon marinus*. Mature larval sea lampreys recover a high degree of their swimming [5] and burrowing [6] abilities and behaviors at around 10 weeks after a complete SCI. In lampreys, regenerated axons make new synapses below the site of a complete SCI [7] and functional recovery is associated with the regeneration of descending axons [8]. However, even in lampreys, regeneration of descending axons is not complete [9] and anatomical and functional plastic changes in spinal cord circuits must occur to achieve the high degree of functional recovery observed after SCI [8], [10], [11], [12], [13], [14], [15]. Thus, lampreys offer a model to identify the genes and signaling pathways that promote or restrict spontaneous and successful axon regeneration after SCI in vertebrates. Research in this area in lampreys has mainly focused on the analysis of the giant reticulospinal neurons of the sea lamprey brainstem [4], [16], [17], [18]. These giant neurons are very interesting as a model because they can be identified individually from brain to brain and because they show very different regenerative abilities after a complete SCI, which has led to the discovery of several molecules and signaling pathways regulating neuronal survival and axon regeneration (e.g. cAMP: [19], [20], [21]; GABA: [22], [23], [24]; Wnt: [25]; Neogenin: [26]; serotonin: [27]; taurine: [13]; gamma-secretase: [14]; RhoA: [28]; chondroitin sulfate proteoglycans [29]). However, the presence of these giant reticulospinal neurons (e.g., the Mauthner cell) is characteristic of fish and amphibian species and similar giant descending neurons are not found in mammalian brains. In addition, the study of the regeneration of these neurons in lampreys usually involves the use of tract tracing methods to label/visualize the giant regenerated neurons. Tracer application increases experimental timing and involves causing a second complete SCI below the site of the original injury and several days of tracer incubation to label the regenerated neurons.

In recent years, us and others have initiated the characterization of the changes and recovery of neurochemically-distinct neuronal populations with small caliber axons after a complete SCI in lampreys by using immunofluorescence methods (serotonin: [30]; dopamine: [10]; glutamate: [11], [31]; GABA: [12], [31]; glycine: [31], [32]). These studies revealed some of the anatomical plastic changes that occur in these neurotransmitter systems in the spinal cord of sea lampreys during recovery from a complete SCI. However, the presence of intrinsic spinal cord cells expressing these classic monoaminergic or aminoacidergic neurotransmitters did not allow to differentiate between the regeneration of descending axons coming from the brain and the contribution of axons from intrinsic neurons by using only immunofluorescence methods. For example, we had to use double tracer labeling to show that descending serotonergic axons of lampreys regenerate spontaneously after a complete SCI [33]. Interestingly, we have recently discovered the presence of a descending system of neurons that express the neuropeptide corticotropin-releasing hormone (CRH) in the sea lamprey brain [34]. CRH is a neuropeptide of interest in the SCI context because descending CRH projections from the Barrington’s nucleus (pontine micturition center) to the spinal cord control micturition behavior in mammals [35], [36]. Specifically, CRH from Barrington’s nucleus spinal cord-projecting neurons inhibits micturition [36], [37], [38]. Restoration of urination control is one of the main aims in SCI patient management [39]. Importantly, CRH projections from the Barrington’s nucleus are only minimally recovered in lumbosacral levels 4 weeks after an incomplete spinal cord transection at T8/T9 (thoracic level) in rats [40]. In the spinal cord of mature larval sea lampreys, CRH neurons are only present in the most rostral spinal cord, close the rhombencephalon/spinal cord transition [34]. At the level of the 5th gill opening or at more caudal spinal cord levels (midbody region or most caudal spinal cord) only CRH descending axons are present and there are no intrinsic spinal cord CRH [34]. These descending CRH axons originate in the isthmic and medial reticular nuclei of the rhombencephalon and in the nucleus of the medial longitudinal fascicle of the caudal diencephalon as shown by combined tracer application at the level of the 5th gill and CRH immunofluorescence. Thus, this newly discovered CRH descending system offers the possibility of analyzing the axon regenerative ability of a neurochemically-distinct neuronal system by using immunofluorescence methods.

Here, we used an antibody that we previously generated against the sea lamprey mature CRH peptide [34] to study the regeneration of CRH descending axons after a complete SCI at the level of the 5th gill in larval sea lampreys. Our results reveal a full regeneration of the descending CRH innervation below the site of injury (at the level of the 6th gill opening) 10 weeks after the complete SCI. Double labeling with a pre-synaptic marker showed that regenerated CRHergic axons probably re-established synaptic contacts below the site of injury. This work provides a new model to analyze spontaneous axon regeneration in a specific neuropeptidergic system by using a simple immunofluorescence method in lampreys.

## 2 Material and methods

### 2.1 Animals and complete SCI

Mature and developmentally stable larval sea lampreys, *Petromyzon marinus* L. (n = 28 animals in total; between 100 and 150 mm in body length, 5 to 7 years of age), were used in this study. Sex of the animals cannot be determined at this larval stage and larvae were randomly assigned to the control or injury groups. Animals were anaesthetized with 0.1 % MS-222 (Sigma, St. Louis, MO, USA) in lamprey Ringer solution (137 mM NaCl, 2.9 mM KCl, 2.1 mM CaCl2, 2 mM HEPES; pH 7.4) before all experimental procedures and euthanized by decapitation at the end of the experiments. All experiments were approved by the Bioethics Committee of the University of Santiago de Compostela and the Xunta de Galicia (Galicia, Spain; license number 15012/2020/011) and were performed in accordance with European Union and Spanish guidelines on animal care and experimentation.

Complete spinal cord transections were performed as previously described [41]. Briefly, the rostral spinal cord was exposed from the dorsal midline at the level of the 5th gill opening by making a longitudinal incision with a scalpel (#11). A complete spinal cord transection was performed with Spring scissors (Fine Science Tools, Heidelberg, Germany; Cat#15024-10). SCI surgeries were always performed by the same experimenter. Completeness of the injury was assessed under the microscope by visualizing the spinal cord cut ends at the time of surgery and was confirmed 24 h after surgery by checking that there were no caudally propagating movements below the 5th gill. Injured animals were allowed to recover in individual fresh-water tanks at 19.5 °C for 4 or 10 weeks.

### 2.2 Behavioral analyses

Behavioral recovery (swimming ability) of 10 weeks post-lesion (wpl) animals was analyzed based on the study of Ayers (1989) [42] and following the protocol of Hoffman and Parker (2011) [43]. This qualitative assessment of locomotor function was made from video recordings of 5 min (camera: Panasonic Full-HD HC-V110). The animals were placed in a plastic aquarium (36 × 23 × 10.5 cm) and swimming activity was initiated by lightly pinching the tail of the animal using a pair of forceps. Locomotor recovery of the animals was categorized in a scale of 1 to 6 [42], [43]. Animals in stages 5 to 6 correspond to animals in which regeneration of axons through the site of injury has occurred based on activity evoked by stimulation across the lesion site in the isolated spinal cord [43]. Two blinded experimenters independently evaluated each 10 wpl animal. Based on both analyses, a mean value of locomotor recovery was obtained for each 10 wpl animal.

### 2.3 Immunofluorescence

**Fig. 1:**
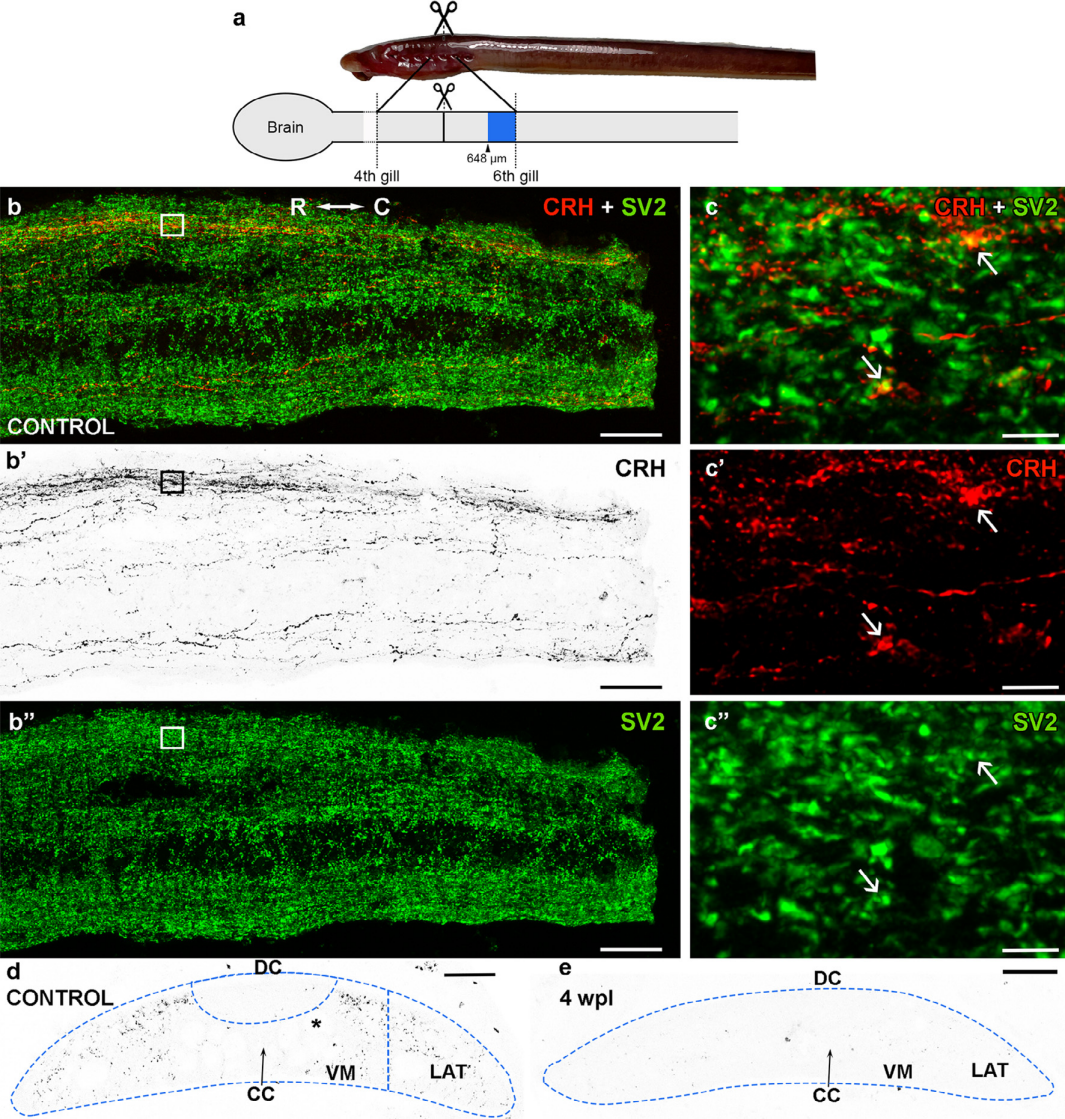
CRH-ir axons at the level of the 5th and 6th gill openings are of descending origin. **a.** Lateral view of a larva and schematic drawing of a lateral view of the brain/spinal cord indicating the site of injury (scissors) and area of analysis (blue). **b-b**″**.** Sagittal spinal cord sections double labeled with CRH (red or B&W) and SV2 (green) antibodies showing the longitudinal trajectory of descending CRH-ir axons. Note that these are more abundant in the dorsal portion of the spinal cord (b-b’). **c.** Details of CRH-ir axons (square in b-b″) showing co-localization with SV2 (arrows). **d.** Transverse spinal cord section of a control un-injured animal showing the distribution of CRH-ir axons. **e.** Photomicrograph showing the lack of CRH-ir axons below the site of injury in a 4 wpl animal (e). Photomicrographs b’, d and e with red fluorescence were converted to B&W. Photomicrographs c-c″ were taken with lightning adaptive deconvolution. Abbreviations: C: caudal, CC: central canal, DC: dorsal column, LAT: lateral region, R: rostral, VM: ventromedial region. Scale bars, 50 µm (b-b″), 3 µm (c-c″), 75 µm (d, e). (For interpretation of the references to colour in this figure legend, the reader is referred to the web version of this article.)

For immunofluorescence experiments the spinal cord tissue between the 4th and 6th gill openings (Fig. 1a) was dissected out and fixed by immersion in 4 % paraformaldehyde (PFA) in 0.05 M Tris-buffered saline pH 7.4 (TBS) for 6 h at 4 °C. The spinal cord samples were then rinsed in TBS, cryoprotected with 30 % sucrose in TBS, embedded in Tissue Tek (Sakura, Torrance, CA, USA), frozen in liquid nitrogen-cooled isopentane, and cut serially on a cryostat (18 µm thickness) in sagittal or transverse planes. Sections were mounted on Superfrost® Plus glass slides (Menzel).

The spinal cord sections were then incubated with a combination of a purified rabbit polyclonal anti-sea lamprey CRH antibody (dilution 1:1500; [34]; RRID: AB_2923134) and a mouse monoclonal anti-synaptic vesicle glycoprotein 2 (SV2) antibody (1:250; Developmental Hybridoma Bank, University of Iowa, Iowa, USA; RRID: AB_2315387) at room temperature overnight. The sections were then rinsed in TBS and incubated for 1 h at room temperature with a combination of a Cy3-conjugated goat anti-rabbit antibody (1:500; Millipore, Burlington, MA; Cat# AP132C; RRID: AB_92489) and a FITC-conjugated goat anti-mouse IgG antibody (1:200; Invitrogen, Carlsbad, CA; Cat# F2761; RRID: AB_2536524). All antibodies were applied in a solution containing 15 % normal goat serum and 0.2 % Triton. Sections were finally rinsed in TBS and distilled water and mounted with Mowiol® (Sigma).

### 2.4 Specificity of primary antibodies

The anti-CRH antibody was generated against the mature sea lamprey CRH peptide (CSDEPPISLDLTFHLLREVLEMAKAEQLAQQAHTNRQIMENI-NH2) modified with the addition of a cysteine (C) residue to the *N*-terminus. The peptide was synthesized by Biomedal (Sevilla, Spain) and the cysteine modification was introduced to allow coupling to key limpet hemocyanin (KLH). A rabbit (8 weeks old, New Zealand White) was first immunized subcutaneously with the peptide-KLH conjugate (400 mg) emulsified in Freund’s complete adjuvant. After 4 weeks, the rabbit was immunized once a week for 2 weeks via intramuscular injection of the peptide-KLH conjugate (200 mg) emulsified in Freund’s complete adjuvant. Pre-immune (negative control with no anti-CRH antibodies present) and post-immunization bleeds were collected. 2.5 mL of antiserum from the final bleed were purified using a protein A-Sepharose column (GE Healthcare, Little Chalfont, UK).

The anti-serum and the purified fraction of anti-CRH antibody were tested for specificity with an ELISA using standard methods [34]. The antiserum, the purified antibody (at concentrations from 1:1000 to 1:100000) and the pre-immune serum (1:1000) were tested against the synthetic CRH peptide without KLH (at a concentration of 1 mM) in the ELISA. Both the antiserum and the purified fraction gave a positive signal at all the tested concentrations, while the very low signal registered with the pre-immune serum was the same as the one obtained in the negative controls with saline only [34]. The pattern of CRH-immunoreactive (-ir) neuronal populations in the central nervous system matches the pattern of *CRH* transcript expressing populations seen by in situ hybridization (see, for example, the magnocellular preoptic nucleus (mPO) labelled with *CRH* in situ hybridization in Fig. 3b-d and with CRH immunofluorescence in Fig. 5a-b of [34] or the posterior entopeduncular nucleus (PEN) labelled with *CRH* in situ hybridization in Fig. 3f and with CRH immunofluorescence in Fig. 5e of [34]), which further supports the specificity of the antibody for this neuropeptide.

The SV2 antibody was made against purified synaptic vesicles from the electric ray [44]. The SV2 antibody recognizes all 3 SV2 isoforms (A to C) and labels synaptic vesicle clusters in all tested vertebrates, including lampreys [21], [45], [46], [47], [48].

### 2.5 Imaging and quantification

Images of fluorescent labelled sections were taken with the Leica TCS-SP2 or with the Leica Stellaris 8 confocal microscopes using blue and green excitation lasers. Confocal optical sections were taken at steps of 2.5 (Leica TCS-SP2) or 0.7 (Leica Stellaris 8) μm along the z-axis when using the 20x objective and at steps of 0.3 μm when using the 40x or 63x objectives. Lightning adaptive deconvolution was used to obtain maximum resolution [49] in double labeling images taken with the Leica Stellaris 8 confocal microscope (40x and 63x objectives). Collapsed images of the whole spinal cord sections (18 μm) were obtained with the LITE or LAS X software (Leica, Wetzlar, Germany). For figure preparation, and always after profile quantifications, contrast and brightness of the images were minimally adjusted using Adobe Photoshop 2021 (Adobe, San Jose, CA, USA). Some photomicrographs with red fluorescence presented in the figures were converted to B&W. Schematic drawings were also generated with Adobe Photoshop 2021.

The number of CRH-ir axonal profiles was quantified in confocal transverse spinal cord sections using the Feature J plugin in the Fiji software, as previously described [31]. Briefly, the confocal images from control and injured animals were always taken with the same microscope (Leica TCS-SP2; 20x objective) and software parameters. A threshold was established to have the most accurate images when converting them to binary B&W images for profile quantification. We quantified the number of CRH-ir profiles in 1 out of every 4 consecutive sections starting from the spinal cord at the level of the 6th gill opening and moving rostrally. A total of 9 sections were analyzed per animal covering 648 µm of spinal cord caudal to the site of injury (Fig. 1a). The mean number of positive CRH-ir profiles per section was obtained for each animal based on the results from the 9 sections. Each dot in the graph represents one animal.

### 2.6 Statistical analyses

Statistical analyses were performed with Prism 9 (GraphPad software, La Jolla, CA, USA). Normality of the data was determined with the D'Agostino-Pearson test. To determine significant differences (p ≤ 0.05) between control and 10 wpl animals, a two tailed Student’s (unpaired) *t*-test was used (normally distributed data). A minimum of 10 animals were included in each group (control and 10 wpl) and they came from 3 different batches. Control and 10 wpl animals were always processed in parallel for each batch.

## 3 Results and discussion

As indicated in the introduction, we have recently generated an antibody against the sea lamprey CRH mature peptide, which allowed us to study the organization of the CRHergic system in mature larval sea lampreys [34]. In the spinal cord, CRH-ir neurons are only present in the most rostral spinal cord (close to the obex). At the level of the 5th gill opening, we only observed the presence of CRH-ir axons ([34]; Fig. 1). Tract-tracing experiments applying a neuronal tracer at this spinal cord level revealed that these descending axons originate from CRH-ir neurons of the isthmic and medial reticular nuclei of the rhombencephalon and of the nucleus of the medial longitudinal fascicle of the caudal diencephalon [34]. Here, we first performed double immunofluorescence experiments in spinal cord sections of non-injured animals at the level of the 6th gill opening. Sagittal spinal cord sections revealed a conspicuous system of descending CRH-ir axons coursing with longitudinal trajectories in the spinal cord (Fig. 1b-b″). As revealed by sagittal sections, these descending axons were more abundant in the dorsal portion of the spinal cord (Fig. 1b-b″). Double labeling with antibodies against the synaptic vesicle marker SV2 revealed colocalization of CRH and SV2 immunoreactivities in the same axonal profiles, which indicates that CRH-ir descending fibers establish synaptic contacts in their course along the spinal cord (Fig. 1c-c″). Transverse spinal cord section at the same level showed that most of the CRH-ir axons navigate the spinal cord tissue in its dorsolateral region, with some axons also found in the ventromedial region (Fig. 1d). No CRH-ir axons were observed in the dorsal column (Fig. 1d). As a way to confirm that all these CRH-ir axons are of descending origin and that they degenerate after axotomy, we first analyzed 4 wpl animals after a complete SCI at the level of 5th gill. The idea is that ascending axons could remain caudally to the site of injury, but descending axons disconnected from the cell bodies should degenerate. As can be observed in transverse sections at the level of 6th gill, the spinal cord of 4 wpl animals is completely devoid of CRH-ir axons below the site of injury (Fig. 1e). Overall, our previous and present results show that all spinal cord CRH-ir axons present at the level of the 5th and 6th gill openings are of descending origin and that they degenerate caudally to the site of the complete SCI within the first 4 wpl.

Based on these results we decided to analyze the regenerative ability of axotomized descending CRH-ir axons. For this we selected the 10 wpl time point because previous work has shown that at this time point larval lampreys usually show a high degree of functional recovery [6], [50], [51] and axon regeneration from descending giant neurons [12], [52], [53] after a complete SCI at the level of the 5th gill. All the 10 wpl larvae in which we quantified the regeneration of descending CRH-ir axons (see below) showed a minimum degree of functional recovery of 4 in the Ayer’s Scale (mean value of 4.9 ± 0.2414; 55 % of the animals had a degree of recovery between 5 and 6 in the Ayer’s scale). At stage 4, rostral and caudal activity becomes coordinated so that propagating waves travel along the length of the body (suggestive of the recovery of intersegmental coordination due to axonal regeneration). However, stage 4 animals, as compared to stage 5/6 animals, typically exhibit a bilateral asymmetry of curvature on one side of the body during swimming, which leads to an inability to maintain a normal dorsal-side-up orientation all the time while swimming.

**Fig. 2:**
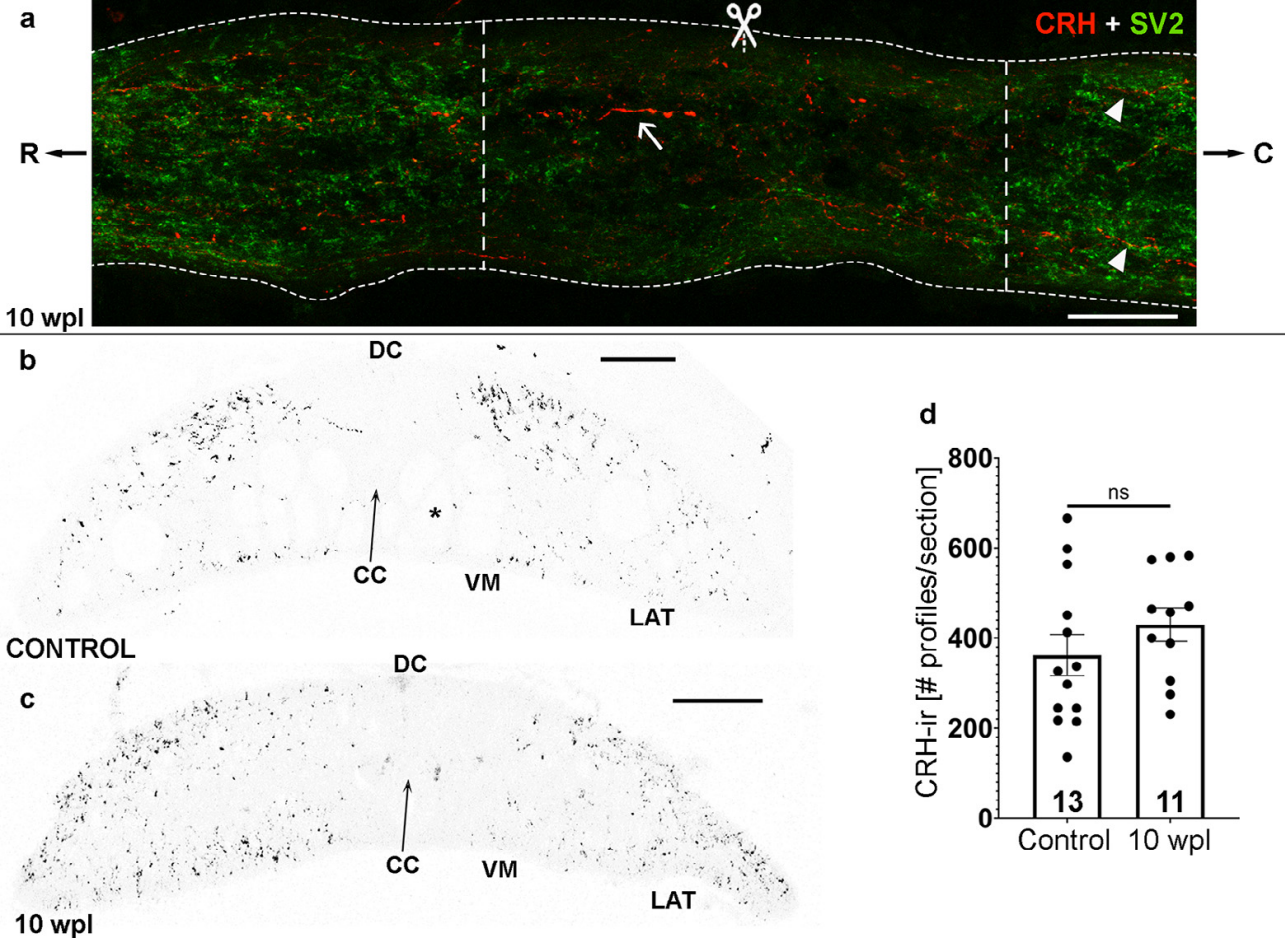
Full regeneration of descending CRH-ir axons at 10 wpl **a.** Sagittal section of the SCI site (level of the 5th gill; scissors) of a 10 wpl animal double labelled with CRH and SV2 antibodies showing the low level of SV2 expression. The scar tissue around the site of injury is indicated with vertical dashed lines (note that this region shows a disorganized tissue structure and lack of strong SV2 immunoreactivity). Regenerated CRH-ir axons crossing the injury site are indicated with and arrow. Arrowheads indicate regenerated axons in the region caudal to the injury site. **b, c.** Transverse spinal cord sections of un-injured (b) and 10 wpl (c) animals at the level of the 6th gill showing that numbers of CRH-ir profiles (axons) in 10 wpl animals were not significantly different to those of controls (see graph in d, each dot represents 1 animal)**.** Photomicrographs b and c with red fluorescence were converted to B&W. For abbreviations, see Fig. 1 legend. Scale bars, 50 µm (a), 75 µm (b, c). (For interpretation of the references to colour in this figure legend, the reader is referred to the web version of this article.)

**Fig. 3:**
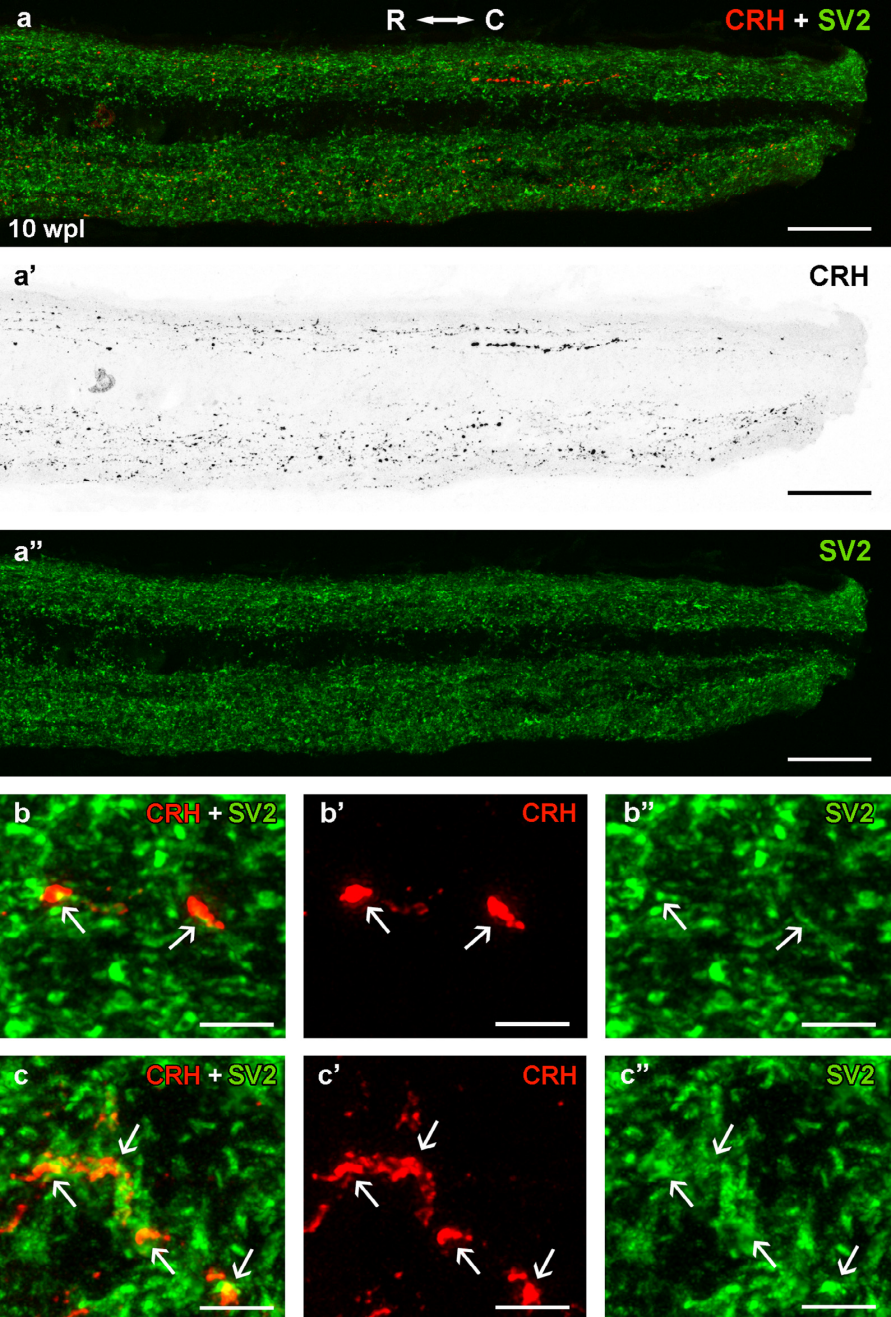
Photomicrographs showing the presence of regenerated CHR-ir axons in the spinal cord (level of the 6th gill) at 10 wpl. **a-a**″**.** Sagittal spinal cord sections double labeled with CRH and SV2 antibodies. **b-c**″**.** Details of regenerated CRH-ir axons in sagittal (b-b″) and transverse (c-c″) spinal cord sections showing the co-localization of CRH and SV2 immunoreactivities (arrows). Photomicrograph a’ with red fluorescence was converted to B&W. Photomicrographs b-b″ and c-c″ were taken with lightning adaptive deconvolution. For abbreviations, see Fig. 1 legend. Scale bars, 50 µm (a-a″), 3 µm (b-b″, c-c″). (For interpretation of the references to colour in this figure legend, the reader is referred to the web version of this article.)

Sagittal sections at the site of SCI (level of the 5th gill) in 10 wpl animals revealed the presence of regenerated CRH-ir longitudinal axons crossing the site of injury and reinnervating the caudal spinal cord (Fig. 2a). The site of injury is clearly identified by the disorganization of the regenerated tissue surrounding the injury and because of the lack of strong SV2 labeling in this region (Fig. 2a). We quantified the degree of axonal regeneration of CRH-ir axons in transverse sections of the spinal cord at the level of the 6th gill (caudally to the site of injury), which revealed a full regeneration of the number of CRH-ir axonal profiles as compared to control non injured animals (control: 362.3 ± 45.79, 10 wpl: 429.9 ± 37.18; Fig. 2b-d). Numbers of CRH-ir profiles in 10 wpl animals were not significantly different to those of controls (p = 0.2756; Fig. 2b-d) and we even observed a tendency for a higher number of axonal profiles in regenerated animals. Double labeling of these regenerated CRH-ir axons with anti-SV2 antibodies in spinal cord sections at the level of the 6th gill revealed colocalization of CRH and SV2 immunoreactivities, which suggests the presence of new pre-synaptic vesicle clusters and synapse regeneration in descending CRH-ir axons below the site of injury (Fig. 3). Therefore, our results show that following a complete SCI in lampreys there is a full regeneration of the descending CRHergic system, at least up to the level of the 6th gill. Regeneration of CRH-ir axons occurs in lampreys with a high degree of functional recovery (Ayer’s test) and these regenerated axons not only regrow through the site of injury and for some millimeters caudally, but also re-establish pre-synaptic contacts with targets below the site of injury. In contrast, CRH projections from the Barrington’s nucleus are only partially recovered in lumbosacral levels 4 weeks after an incomplete spinal cord transection at T8/T9 (thoracic level) in rats [40], which parallels bladder malfunction in these animals. Thus, our work provides a new model to understand spontaneous and successful axonal regeneration in a neurochemically-distinct neuronal population in lampreys, which in the past has mainly relied on the analysis of giant descending neurons by using tracer applications. This is important because neurochemically-distinct neuronal populations can have very different regenerative abilities as shown, for example, with the unusual regrowth capacity of serotonergic axons after injury in mammals [54], [55]. Lampreys offer a model to understand the molecular pathways that specifically promote de regrowth and functional regeneration of descending CRH axons.

Interestingly, *in vitro* work has also shown that CRH can promote axonal outgrowth from rat dorsal root ganglion cell cultures by promoting the release of BDNF from microglial cells [56]. In addition, a recent study analyzing transcriptomic data from contusion and transection SCIs in rats has also revealed a significant increase in CRH levels after SCI [57]. Thus, it would be of interest to study the effects that CRH might have *in vivo* in lampreys on axonal regeneration (CRHergic or other axons) after SCI and our work provides a model for future molecular studies on axon regeneration in the lamprey model.

## CRediT authorship contribution statement

**Laura González-Llera:** Investigation, Methodology, Writing – review & editing. **Daniel Sobrido-Cameán:** Investigation, Methodology, Writing – review & editing. **Gabriel N. Santos-Durán:** Investigation, Methodology, Writing – review & editing. **Antón Barreiro-Iglesias:** Conceptualization, Writing – original draft, Supervision, Funding acquisition.

## Declaration of Competing Interest

The authors declare that they have no known competing financial interests or personal relationships that could have appeared to influence the work reported in this paper.
